# Tennessee Medical Laws

**Published:** 1889-06

**Authors:** Frank Trester Smith

**Affiliations:** Chattanooga, Tenn.


### TENNESSEE MEDICAL LAWS.

*Editor Atlanta Medical and Surgical Journal:*

At the past session of the Legislature, bills regulating the practice of Dentistry and Pharmacy were defeated. The bill regulating the practice of Medicine passed. The following is a synopsis of the bill:

There is a State Board of Examiners of six members, not more than four to be of the same "school" of medicine. The members of the Board are appointed by the Governor. No one is allowed to practice without a certificate from the Board, under penalty of $100 for first, and $200 for each subsequent offense. All persons in practice at time of passage of act given certificate on presenting proof of this fact. Those hereafter desiring to practice must present diploma from a medical college in good standing, or pass examination in Anatomy, Physiology, Chemistry, Pathology, Surgery and Obstetrics. The fee for examination is ten dollars, five of which is refunded if candidate fails to pass. The fee for a certificate is one dollar. The members are allowed ten dollars per day, with hotel and traveling expenses, for their services. They meet three times a year—once in each grand division of the State. Itinerants are to pay $100 per month for the privilege of practicing. This act does not apply to midwives. Act goes into effect sixty days after passage.

FRANK TRESTER SMITH, M. D.

*Chattanooga, Tenn., April 22, 1889.*

